# Bilateral superficial temporal vein thrombosis after acute carbon monoxide poisoning and prolonged immobilisation: a case report

**DOI:** 10.1093/omcr/omad117

**Published:** 2023-12-19

**Authors:** Rémy Hamdan, Benoît Bach, Jacques Asdrubal, Anne Laure Baldassini

**Affiliations:** Department of Vascular Medicine, Macon Hospital Centre, Mâcon, France; Department of Internal Medicine, Macon Hospital Centre, Mâcon, France; Department of Emergency Medicine, Macon Hospital Centre, Mâcon, France; Department of Vascular Medicine, Macon Hospital Centre, Mâcon, France

## Abstract

A prolonged stay on the ground after acute carbon monoxide poisoning (COP) is a high-risk situation for venous thromboembolism (VTE), but unusual-site venous thrombosis is rare in this setting. An 81-year-old woman with no personal or family history of VTE who lied on the ground for several hours following massive COP had painful and oedematous temples, so a Doppler ultrasound was prompted and revealed a bilateral superficial temporal vein (STV) thrombosis. There was no heart failure, trauma, inflammatory disease, infection, or vascular malformation. The thrombosis regressed on fondaparinux 2.5 mg given as a daily subcutaneous injection for 45 days. Our observation emphasizes the need to look not only for arteritis but also for venous thrombosis before any temporal pain. STV thrombosis has been reported four times to date. We report the first case of bilateral STV thrombosis in the setting of massive COP and prolonged immobilisation in an elderly patient.

## INTRODUCTION

As carbon monoxide (CO) is tasteless, odourless and colourless, carbon monoxide poisoning (COP) may be prolonged, lead to non-specific symptoms such as headaches, dizziness, or confusion, and cause the elderly to fall and lie on the ground for a long time [1]. This situation carries a high risk of venous thromboembolism (VTE), as its incidence is increased in cases of prolonged immobilisation [2], advanced age [3], or COP [4]. However, this enhanced risk concerns the occurrence of lower limb deep vein thrombosis or pulmonary embolism, with cases of venous thrombosis in unusual sites being very rare. We report a hitherto unseen case of bilateral thrombosis of the superficial temporal vein (STV), which complicates a long-lasting lie on the ground after a fall in a CO-poisoned elderly person.

## CASE REPORT

A visitor discovered an 81-year-old woman on the floor of her home. The firefighters detected the carbon level in the atmosphere at 180 ppm and 234 ppm in the exhaled air. At the emergency room, her blood pressure was 143/92 mmHg, her pulse rate was 97 beats per minute, her body temperature was 35.7°C, her weight was 71 kg, and her Glasgow Coma Scale score was 13 (opening eye in response to voice, confused and disoriented but able to answer questions). She had been missing for two days and had clearly been on the ground for many hours. Her medical history included a surgically removed left breast carcinoma 30 years ago and a double superior vena cava (SVC). Her family had never had a VTE episode, and she had never had one herself. The temples were swollen and painful, and palpation of the scalp over the left parietal bone exacerbated the pain. The temporal pulses were perceived, and sinus palpation was painless. Upper respiratory tract examination, cardiopulmonary auscultation, and palpation of the hand-and-neck nodes were normal.

**Figure 1:**
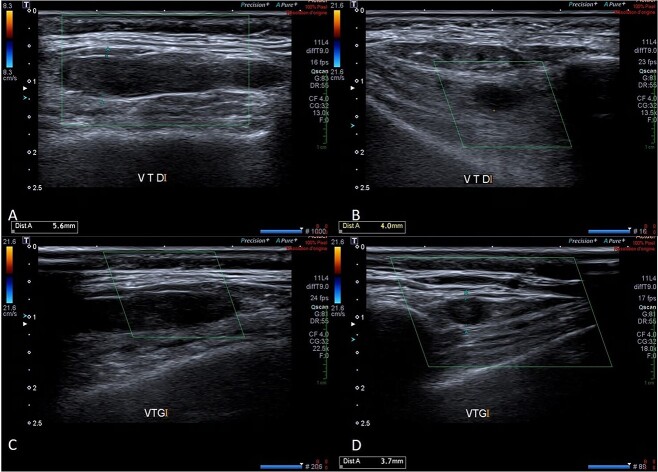
Doppler ultrasound images taken during temples examination. A: Longitudinal section of thrombus approximately 30 mm long in the right superficial temporal vein. B: Transversal section of the thrombus in the right superficial temporal vein, 4 mm thick. C: Longitudinal section of a thrombus over 20 mm long in the left superficial temporal vein. D: Cross-section of the thrombus in the left superficial temporal vein, thickness measured at 3.7 mm.

**Figure 2:**
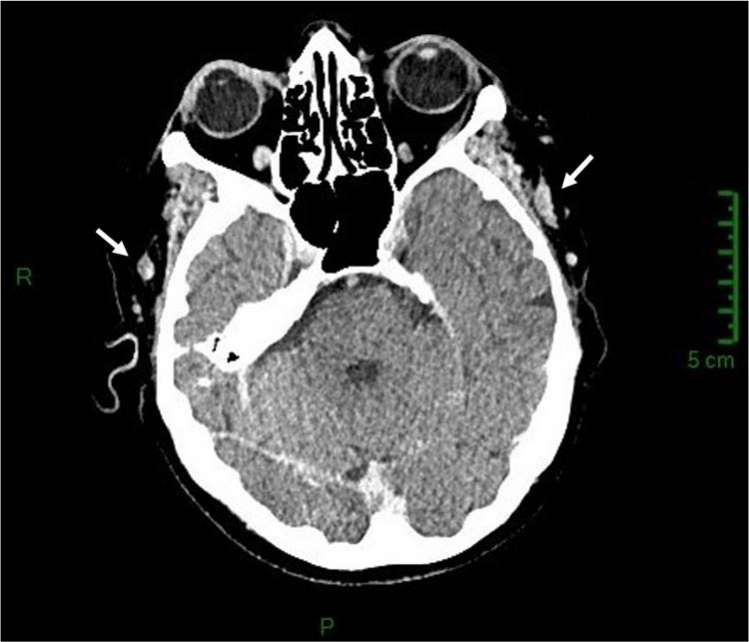
Axial section of the brain computed tomography scan showing thrombosis of the two superficial temporal veins (arrows).

The carboxyhaemoglobin was 13% (normal range 0.5–1.5%), and the arterial blood gas results showed hypoxia at 31.3 mmHg (normal 35–40 mmHg), partial pressure of carbon dioxide at 38.9 mmHg (normal 36–44 mmHg), oxyhaemoglobin at 61% (normal 68–73%), oxygen saturation at 70.6% (normal 68–73%), and lactates at 2.1 mmol/l (normal 0.6–1.4 mmol/l). Oxygen therapy and an intravenous infusion of isotonic saline were started immediately. The white blood cell count was 14.34/l (normal 3.8–9.1/l), including neutrophils at 12.81/l (normal 1.5–5.40/l); C-reactive protein (CRP) was 129 mg/l (normal: less than 5 mg/l); creatine phosphokinase (CPK) was 968 international units (IU)/l (normal: less than 170 UI/l); troponin was 0.26 ng/l (normal: less than 0.10 ng/l); creatinine was 83 mol/l (estimated glomerular filtration rate 57 mL/min); and the N-terminal prohormone of brain natriuretic peptide (NT-proBNP) was 13 294 ng/L (normal: less than 125 ng/l). The electrocardiogram showed sinus rhythm with a normal ST-segment. Doppler ultrasound (DUS) revealed a thrombosis of the right STV extending to its junction with the maxillary vein and a thrombosis of the left STV beginning in the parietal scalp veins (Figure 1), whereas the temporal arteries had normal, regular walls and the jugular veins were thrombosis-free. A body’s computed tomography scan showed no sign of malignancy or concomitant VTE, ruled out cerebral venous thrombosis, and confirmed bilateral STV thrombosis with extension to the scalp on the left (Figure 2). A daily subcutaneous injection of 2.5 mg fondaparinux was therefore initiated for 45 days. A body’s fluorodeoxyglucose-positron emission tomography (PET) scan revealed no hypermetabolism suggestive of vasculitis, chronic inflammatory rheumatism, or malignancy, and a transthoracic echocardiogram was normal. After a home’s technical inquiry, the boiler was brought up to standard, and in-home care services were solicited. The patient rapidly ceased to be confused; her WBC count, troponin, CRP, and CPK returned to normal; her blood creatinine decreased; and, after 4 days of uneventful hospitalization, she was discharged. At a one-month follow-up consultation, while the neurological examination was normal, the patient was complaining of memory loss and anhedonia. At the follow-up DUS examination six weeks later, the thrombi had completely regressed, so anticoagulation was discontinued.

## DISCUSSION

STV thrombosis is a rarely reported condition that masquerades as cranial giant cell arteritis (GCA) [5]. In a clinical observation, a patient with histology-proven GCA experienced a right STV thrombosis that mimicked a disease relapse [6]. In a second reported case, a GCA preceded a bilateral STV thrombosis that was attributed to a GCA’s active phase and was successfully treated with anticoagulation [7]. In another case report, a GCA was suspected in a 75-year-old woman with a painful 20-mm cord of the right temple before a scalp-extended thrombosis of an orbital venous angioma’s draining vein was diagnosed [8]. A right STV thrombosis was reported in a final case report alongside acute bacterial rhinosinusitis [9]. Concerning our patient, the absence of hypermetabolism indicative of vasculitis on PET and the presence of normal temporal arteries on DUS ruled out CGA, and there was no evidence of a concomitant infectious condition or a prior vascular malformation.

The patient did not benefit from hyperbaric oxygen therapy because the NT-proBNP level was very high. Given that the clinical signs of cardiac dysfunction were absent, NT-proBNP is eliminated exclusively by the kidneys, and its level decreased with renal function’s recovery, we believe that the rhabdomyolysis-induced acute kidney failure provoked such a high NT-proBNP’ level and that heart failure never occurred. An increase in pressure in the right heart chambers secondary to left-heart decompensation cannot therefore have contributed to the STV thrombosis. Additionally, given the STV thrombosis was bilateral, long-term mechanical compression was also unlikely to be the culprit, and our patient’s duplicate SVC is not known to contribute to thrombotic events. Several months after her COP, the patient reported memory and mood disorders, suggesting that the intoxication had been massive. Given the *a priori* high exposure to CO and the fact that a causal relationship between COP and prolonged immobilisation is not certain, it would be tempting to establish a direct link between COP and the thrombotic event. However, the mechanisms behind a VTE’s increased risk following a COP are unclear [10], and such a hypothesis does not explain the thrombosis site’s unusualness. In the absence of a VTE history, recent surgery, obesity, cancer, heart failure, respiratory failure, or inflammatory disease, as well as any argument for a local cause of venous thrombosis of an infectious, mechanical, or inflammatory nature, bilateral STV thrombosis appeared to be induced by prolonged immobilisation, advanced age, and acute medical illness (massive COP in the event we report).

Based on our information, acute bilateral STV thrombosis complicating a long-lasting lie on the floor in the setting of COP has never been reported. In the case of temple pain in an elderly patient with inflammation, if DUS does not provide any evidence of GCA, it may reveal one of its differential diagnoses, such as STV thrombosis.

## Data Availability

The data supporting this case report is available from the corresponding author on reasonable request.

## Notes

Listed authors:

Benoît Bach – Service de Médecine Interne, Centre Hospitalier de Mâcon - 350, Boulevard Louis Escande - 71000 Mâcon - France. Email: bebach@ch-macon.fr. Telephone: +33.3.85.27.53.33.

Jacques Asdrubal – Service d’Accueil des Urgences, Centre Hospitalier de Mâcon -350, Boulevard Louis Escande - 71000 Mâcon - France. Email: jaasdrubal@ch-macon.fr. Telephone: +33.3.85.27.53.17.

Anne Laure Baldassini – Explorations fonctionnelles de Médecine Vasculaire, Centre Hospitalier de Mâcon - 350, Boulevard Louis Escande - 71000 Mâcon - France. Email: al.baldassini@ch-macon.fr. Telephone: +33.3.85.27.59.70.

## References

[1] Fleming J, Brayne C, Cambridge City over-75s Cohort (CC75C) study collaboration. Brayne C, and the Cambridge City over-75s cohort (CC75C) study collaboration. Inability to get up after falling, subsequent time on floor, and summoning help: prospective cohort study in people over 90. BMJ 2008;337:a2227. 19015185 10.1136/bmj.a2227 PMC2590903

[2] Zee AA, Van Lieshout K, Van Der Heide M, Janssen L, Janzing HM. Low molecular weight heparin for prevention of venous thromboembolism in patients with lower-limb immobilization. Cochrane vascular group, editor. Cochrane Database Syst Rev 2017;8:CD006681. 28780771 10.1002/14651858.CD006681.pub4 PMC6483324

[3] Næss IA, Christiansen SC, Romundstad P, Cannegieter SC, Rosendaal FR, Hammerstrøm J. Incidence and mortality of venous thrombosis: a population-based study. J Thromb Haemost 2007;5:692–9. 17367492 10.1111/j.1538-7836.2007.02450.x

[4] Cho Y, Kang H, Oh J, Lim TH, Ryu J, Ko BS. Risk of venous thromboembolism after carbon monoxide poisoning: a Nationwide population-based study. Ann Emerg Med 2020;75:587–96. 31759754 10.1016/j.annemergmed.2019.08.454

[5] Evangelatos G, Grivas A, Pappa M, Kouna K, Iliopoulos A, Fragoulis GE. Cranial giant cell arteritis mimickers: a masquerade to unveil. Autoimmun Rev 2022;21:103083. 35341973 10.1016/j.autrev.2022.103083

[6] Audia S, Falvo N, Leguy-Seguin V, Berthier S, Martin L, Bonnotte B. et al. Scalp vein thrombosis mimicking giant cell arteritis relapse: brief communication. Intern Med J 2011;41:492–5. 21707894 10.1111/j.1445-5994.2010.02396.x

[7] De Coppet H, Lambert M, Charlanne H, Launay D, Morell-Dubois S, Maillard-Lefebvre H. et al. Superficial cranial venous thromboses preceding the diagnosis of giant cell arteritis. J Mal Vasc 2010;35:23–5. 19879706 10.1016/j.jmv.2009.09.001

[8] Maramattom BV . Temporal arteritis or phlebitis? J Neurol Neurosurg Psychiatry 2006;77:673–3. 16614032 10.1136/jnnp.2005.081422 PMC2117428

[9] Huth ME, Caversaccio MD. Thrombophlebitis of the temporal vein as an extracranial complication of acute bacterial rhinosinusitis. Ear Nose Throat J 2015;94:E48–51. 26322458

[10] Xu Y, Li W. Is carbon monoxide poisoning a risk factor or a bystander of deep venous thrombosis? Ann Emerg Med 2020;75:781–2. 32471580 10.1016/j.annemergmed.2020.01.032

